# Essential Roles of PIEZO1 in Mammalian Cardiovascular System: From Development to Diseases

**DOI:** 10.3390/cells13171422

**Published:** 2024-08-26

**Authors:** Chengjiang Jin, Sheng’an Su, Shuo Yu, Yue Zhang, Kaijie Chen, Meixiang Xiang, Hong Ma

**Affiliations:** 1 Cardiovascular Key Laboratory of Zhejiang Province, National Key Laboratory of Vascular Implantable Devices, Department of Cardiology, The Second Affiliated Hospital, School of Medicine, Zhejiang University, Hangzhou 310009, China; 2 Department of Anesthesiology, The Second Affiliated Hospital, School of Medicine, Zhejiang University, Hangzhou 310009, China

**Keywords:** PIEZO1, mechanotransduction, cardiovascular system, development, homeostasis, diseases, chemical therapies

## Abstract

Mechanical force is the basis of cardiovascular development, homeostasis, and diseases. The perception and response of mechanical force by the cardiovascular system are crucial. However, the molecular mechanisms mediating mechanotransduction in the cardiovascular system are not yet understood. PIEZO1, a novel transmembrane mechanosensitive cation channel known for its regulation of touch sensation, has been found to be widely expressed in the mammalian cardiovascular system. In this review, we elucidate the role and mechanism of PIEZO1 as a mechanical sensor in cardiovascular development, homeostasis, and disease processes, including embryo survival, angiogenesis, cardiac development repair, vascular inflammation, lymphangiogenesis, blood pressure regulation, cardiac hypertrophy, cardiac fibrosis, ventricular remodeling, and heart failure. We further summarize chemical molecules targeting PIEZO1 for potential translational applications. Finally, we address the controversies surrounding emergent concepts and challenges in future applications.

## 1. Introduction

Mechanical force is the basis of cardiovascular events, shaping cells and tissues during both development and adult homeostasis, including heart chamber and vascular network formation [1,2]. Even before the circulation is well established, the heart primordium generates mechanical forces. Disruptions in mechanical forces lead to cardiac and vascular dysplasia and later, their structural remodeling and even disease development [3,4]. The process of the cellular perception of external mechanical forces to its corresponding pathophysiological response is known as mechanotransduction, converting extracellular physical–mechanical signals into intracellular biochemical signals. Mechanotransduction is mediated by a wide variety of mechanosensitive channel proteins found in numerous mechanosensitive cells [5]. Various cells within the cardiovascular system express a variety of mechanosensitive (MS) channels from different ion channel families to facilitate the conversion of mechanical forces, including fluid shear, cyclic stretching, and inward pressing [6].

PIEZO is a newly identified mechanosensitive cation channel family that is involved in the transduction of various mechanical signals, including tactile, proprioceptive, and other sensory activities in mammals [7]. There are two isoforms of PIEZO proteins, PIEZO1 and PIEZO2 [7]. Increasing evidence demonstrates that PIEZO1 is widely expressed in various cell types in the mammalian heart and vasculature and plays an essential role in mechanotransduction from development and homeostasis to diseases [8,9]. Although PIEZO2 has been reported to be expressed on pressure receptors, synergizing with PIEZO1 to regulate baroreflex and blood pressure [10], it has been more commonly reported that PIEZO2 is expressed in sensory neurons (including touch neurons, proprioceptors, thermoreceptors, and nociceptors) and in restricted epithelial cells (including Merkel cells and enterochromaffin cells of the intestine) to modulate physiological responses, such as touch, pain and temperature, proprioception, respiration, and digestion [11]. Cardiovascular tissues are not a major region of PIEZO2 expression [11]. To address the translational potential of PIEZO1 in the cardiovascular system, we provide an overview of current research advances in PIEZO1, highlighting its role in cardiovascular development, homeostasis, and diseases. Additionally, we explore a range of PIEZO1 chemomodulators and their therapeutic potentials in cardiovascular diseases. Finally, we discuss the emergent concepts and challenges in PIEZO1 research.

## 2. Structure and Mechanogating Mechanism

**Figure 1:**
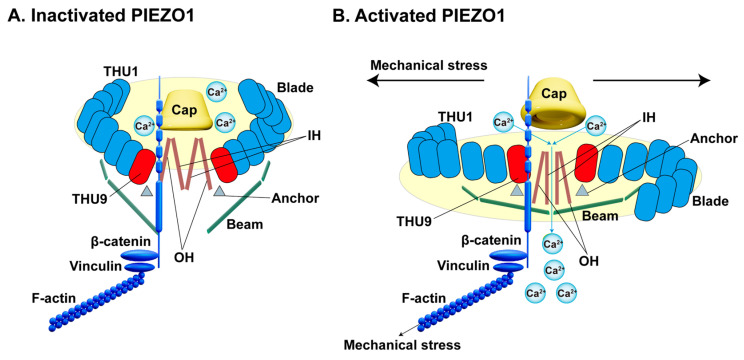
Schematic diagram of PIEZO1 structure and mechanogating mechanism. (**A**) PIEZO1 in a resting state. (**B**) PIEZO1 in an activated state. IH: inner helices, OH: outer helix.

PIEZO1 is a highly conserved protein with a large molecular weight of 300 kDa. Emergent structural data show a unique membrane protein arrangement, which is a three-bladed, propeller-shaped trimer with a curved inverted dome shape in the membrane (Figure 1). Both human and mouse PIEZO1 (hPIEZO1 and mPIEZO1) have high structural homology, containing three subunits with 38 transmembrane (TM) helices each, totaling 114 TM helices, with 4 TM helices forming a single TM helical unit [12]. The outer 36 TM helices form three distinct propeller-like blades that are involved in sensing mechanical stress. The inner OH-CED (C-terminal extracellular domain)-IH-CTD (C-terminal domain) forms a central ion pore, which determines the opening and closing of the PIEZO1 channel. Additionally, three long lever-like beams connect the blade to the central ion pore, effectively translating large changes in the distal blade into small gating changes in the central pore (Figure 1) [13]. This unique lever mechanization structure facilitates cation permeation and the mechanochemical modulation of PIEZO1.

PIEZO1 is present in the plasma membrane of cells and can be activated by various forms of external mechanical forces, such as cell indentation, stretch, shear stress, and substrate mechanics [14,15,16], as well as by intracellular traction [17]. It primarily triggers extracellular Ca^2+^ inward flow and alters intracellular calcium homeostasis (Figure 1) [18]. Two activation modes of PIEZO1 have been proposed (Figure 1). The first is the force-from-lipids mechanism, whereby mechanical stress on the plasma membrane is transmitted to PIEZO1 as membrane tension, leading to PIEZO1 conformational changes [19,20,21]. The second is the force-from-filament mechanism, by which external mechanical stress is sensed by the extracellular matrix (ECM) and intracellular actin cytoskeleton and subsequently indirectly acts on PIEZO1 through the transmission of the cadherin-β-catenin mechanotransduction complex, resulting in conformational changes [22]. But compared to the former, the evidence for this mechanism is still weak and may not be the main mechanism, or these two mechanisms may co-exist and act synergistically.

## 3. PIEZO1 in Cardiovascular Development

PIEZO1 functions as a mechanical stress sensor in the mammalian cardiovascular system and is expressed at various stages of development. It has been found to be expressed in the endothelial cells (ECs) and endocardium of mouse embryos at embryonic day 9–10 (E9–10) [23]. Moreover, postnatally, PIEZO1 is expressed in various types of cells in the cardiovascular system, including vascular endothelial cells (VECs), vascular smooth muscle cells (VSMCs), macrophages (Møs), fibroblasts (FBs), and cardiomyocytes (CMs) [8,9]. Physiological mechanical stress can activate PIEZO1 to promote the development and maintenance of homeostasis in the cardiovascular system. However, when activated by pathological factors or aberrantly expressed, PIEZO1 may contribute to various cardiovascular diseases.

### 3.1. PIEZO1 in Embryos

The complete deletion of PIEZO1 in structure or function (protein mutation or specific PIEZO1 knockout) is lethal to mammalian embryos [23,24]. It was found that while maternal heterozygous mice with a partial deletion of PIEZO1 could survive and give birth, they were unable to give birth to homozygous mice with a complete deletion of PIEZO1 due to the embryonic lethality of the complete deletion of PIEZO1 [23,24]. Interestingly, more in-depth studies have revealed that the progression of the embryo from development to death is surprisingly consistent with the progression of its cardiovascular developmental disorders. This finding seems to imply that the lethality of embryos with a complete deletion of PIEZO1 is attributable to developmental disorders of the cardiovascular system [23,24]. Embryonic mice with a complete deletion of PIEZO1 do not die early in gestation. Prior to E9.5, the cardiovascular system of embryonic mice with a complete deletion of PIEZO1 perceived mechanical stresses, such as blood flow shear, and developed normally, with regular heartbeats and major blood vessel formation and continued embryonic growth and development. However, after E9.5, with a significant decrease in the expression level of PIEZO1 in the cardiovascular system, as well as a decrease in calpain activity, especially in the vascular endothelium and the endocardial lumen, the cardiovascular system of the embryonic mice underwent defects in mechanotransduction, which mainly manifested as impaired angiogenesis and defects in myocardial trabeculae, and the development of the embryos subsequently stagnated. At E10.5, pericardial effusion and defects in blood flow circulation appeared, which in turn led to the failure of embryonic organs, and eventually the mice died in mid-embryo (before E14.5) or early postnatal mortality occurred [23,24]. Moreover, the critical role of PIEZO1 in embryonic development and survival has been corroborated in embryonic stem cell experiments in vitro. After researchers subjected embryonic stem cells to specific PIEZO1 knockouts, the proliferation process of embryonic stem cells was hindered [25]. It is thus evident that PIEZO1 is essential for embryonic developmental survival.

### 3.2. PIEZO1 in Vascular Development

**Figure 2:**
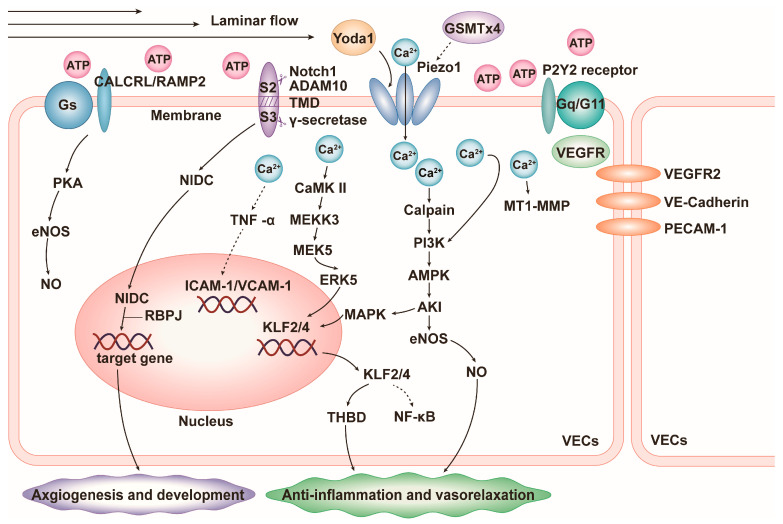
Schematic diagram of the mechanisms by which PIEZO1 promotes vascular development and homeostasis in vascular endothelial cells (VECs). Solid arrows indicate promotion and dashed arrows indicate suppression. ADAM10: Ca^2+^-dependent transmembrane abscission enzyme, ICAM-1: intercellular cell adhesion molecule-1, KLF2/4: kruppel-like factor 2/4, MT1-MMP: membrane-type matrix metalloproteinase 1, PCAM-1: CD31, RBPJ: recombination signal binding protein for immunoglobulin kappa J region, THBD: thrombomodulin, VCAM-1: vascular cell adhesion molecule-1, VE-cadherin: vascular endothelial cadherin, VEGF: endothelial growth factor, VEGFR2: vascular endothelial growth factor receptor 2.

PIEZO1 in VECs plays an essential role in the process of vascular neogenesis and development. Primitive blood vessels are formed by the aggregation of endothelial cell precursors, and PIEZO1 expressed in endothelial cell precursors sensing blood flow shear is activated, which induces the development of primitive blood vessels into mature blood vessels by promoting the differentiation, maturation, and orderly arrangement of VECs. The neovasculature then branches outward to form a complex and organized vascular network [26]. However, after endothelial-specific PIEZO1 knockout or application of the PIEZO1 gene blocker GsMTx4 to inhibit PIEZO1 function, the processes of angiogenesis and development described above are impeded, e.g., in adult animals, endothelial-specific PIEZO1 knockout impedes angiogenesis in the presence of trauma or hindlimb ischemia [23,26,27]. Mechanistically, upon activation of PIEZO1 in VECs by the pressure and shear force of the blood flow, extracellular Ca^2+^ inward flow and intracellular calcium homeostasis are disturbed, thereby activating downstream calcium-dependent calpain [24,28], promoting vascular endothelial growth factor (VEGF)/vascular endothelial growth factor receptor 2 (VEGFR2) to participate in the PI3K/AMPK/AKT/eNOS pathway, and ultimately inducing VECs’ proliferation and differentiation, adhesion turnover, hydrolytic disruption of the cytoskeleton, and rearrangement along the blood flow (Figure 2) [28]. Upon deletion or inhibition of PIEZO1, the phosphorylation of eNOS at the serine 1177 position is reduced and the activity is decreased, which in turn inhibits the proliferation, differentiation, and rearrangement processes of VECs [28]. PIEZO1 can also promote angiogenesis through another classical Notch1 pathway. Activated PIEZO1 mediates the inward flow of Ca^2+^ to activate the metalloproteinase ADAM10 (Ca^2+^-dependent transmembrane abscission enzyme), which participates in the Notch1 pathway as a regulator of S2 cleavage and induces cleavage of the Notch1 S2 site, followed by the γ-secretase that prompts the cleavage of the Notch1 S3 site which leads to the release of N1IDC. N1IDC in turn binds to transcriptional regulators such as recombination signal binding protein for immunoglobulin kappa J region (RBPJ) to upregulate pro-angiogenic gene expression (Figure 2) [29,30]. In addition, PIEZO1-mediated Ca^2+^ inward flow can also link mechanical tugging and pro-vascular factors, such as the activation of matrix metalloproteinase-2 (MMP2) and membrane-type matrix metalloproteinases 1 (MT1-MMPs) in endothelial cells to induce endothelial cell budding (Figure 2) [27] and the activation of CD31 (PCAM-1) to promote bone neovascularization [31].

### 3.3. PIEZO1 in Cardiac Development

During the embryonic or early postnatal period in mice, cardiac development and damage repair are mainly dependent on the proliferation and differentiation of cardiomyocytes [32]. It was found that PIEZO1 is involved in cardiomyocyte differentiation and myocardial vascularization as a way to promote heart development and the repair of damaged myocardium [32]. When cardiac myocardial damage induces a stress response, PIEZO1 can be activated by platelet-rich plasma (PRP) in response to stress, mediating inward Ca^2+^ mobility and affecting IGF-1, VEGF-A/C, and platelet-derived growth factor (PDGF)-AA/AB/BB, as well as EGF from PRP, which in turn induces cellular differentiation, proliferation, and migration to maintain stem cell homeostasis and local angiogenesis, ultimately promoting the repair of damaged CMs [32]. In addition, human umbilical cord mesenchymal stem cells (hUC-MSCs) have been reported that its self-renewal and pluripotency properties allow it to differentiate into cardiomyocytes, which may also promote cardiac development and the repair of damaged myocardium [33]. hUC-MSCs’ ability to differentiate into cardiomyocytes is related to the stiffness of their surrounding matrix. However, it was found that hUC-MSCs were more likely to differentiate into CMs on a soft matrix (13–16 kPa) compared to a rigid matrix (62–68 kPa), which may be related to their low expression of PIEZO1 and integrin β1, as well as Ca^2+^ in the soft matrix environment [33]. Integrin signaling also plays an important role in the cross-talk between PIEZO1 and adhesions. PIEZO1 is located in integrin-mediated adhesion in multiple fibroblast types. PIEZO1 accumulates in a force-dependent manner at the adhesive site and dissociates after the disassembly of integrin adhesions. The localization of PIEZO1 is related to the entry of local Ca^2+^ and adhesion decomposition [34]. These two physiological responses described above have opposite requirements for PIEZO1 expression during cardiac development and repair. PIEZO1 may have different roles in heart development and repair in different cells and in different states, just as the expression levels of PIEZO1 are different in atrial and ventricular cardiomyocytes [35]. Thus, more studies are needed to explore this.

### 3.4. PIEZO1 in Lymphatic Valve and Arteriovenous Valve Development

Lymphatic valves are formed by the proliferation and differentiation of lymphatic endothelial cells, as well as their continuous migratory arrangement, and PIEZO1 is crucial in this process. It has been found that homozygous and compound heterozygous mutations in PIEZO1 leads to congenital lymphatic dysplasia, which may be associated with two variant SNPs (single nucleotide polymorphisms), rs201226914 and rs202127176 [36,37]. The MyoD family inhibitor protein MDFIC, as an auxiliary subunit of PIEZO1, can regulate the inactivation of channels, and this interaction plays a role in lymphatic diseases. The deletion of these two genes can lead to diseases such as defects in lymphatic valve development and lymphatic vessel abnormalities [38,39]. PIEZO1 is also indispensable in the development and formation of arteriovenous valves, and PIEZO1 deficiency leads to defects in the early cardiovascular outflow tract and later aortic valves [40]. By analyzing genomic data from patients with left ventricular outflow tract obstruction (LVOTO), it was found that three novel pathogenic PIEZO1 gene variants (Y2022H, K2502R, and S217L) impede the formation of human mitral and aortic valves [40]. In addition, the common SNP rs2911461 in PIEZO1 was found epidemiologically and genetically to be strongly associated with varicose veins [41,42]. PIEZO1 PTVs (protein truncation variants) occurred significantly more frequently in patients with varicose veins than in those without varicose veins [43]. It has also been found that malformations of the cerebral arteries and veins may be associated with compound heterozygous variants, including PIEZO1 [44].

## 4. PIEZO1 in Cardiovascular Homeostasis and Diseases

PIEZO1 plays a critical role in physiological homeostasis and pathological diseases of the cardiovascular system. During vascular events, PIEZO1 regulates changes in vascular inflammation and blood pressure, and the positive or negative regulatory effects of PIEZO1 often depend on the form and degree of mechanical stress that activates it. Similarly, during cardiac events, PIEZO1, when activated by physiological mechanical stress, often plays a role in maintaining cardiac pumping function and anti-fibrotic effects; however, when abnormally activated by pathological factors, PIEZO1 begins to promote pathological cardiac hypertrophy, cardiac fibrosis, and even heart failure.

### 4.1. Vascular Inflammation

VECs express PIEZO1, which is essential for maintaining the self-anti-inflammatory function of blood vessels. The activation of PIEZO1 in VECs by laminar shear stress results in Ca^2+^ influx and thereby the activation of CaMKII and the downstream MEKK3/MEK5/ERK5 signaling pathway. This induces the upregulation of kruppel-like factor (KLF2 and KLF4), which subsequently increases the expression of anti-inflammatory factors (eNOS and THBD) and inhibits the activation of pro-inflammatory factor (nuclear factor-κ-gene binding (NF-κB)), exerting an anti-inflammatory effect (Figure 2) [45]. Additionally, the activation of PIEZO1 in human umbilical vein endothelial cells under static conditions or under laminar flow shear stress inhibits the tumor necrosis factor-α (TNF-α)-induced expression of intercellular cell adhesion molecule-1 (ICAM-1) and vascular cell adhesion molecule-1 (VCAM-1), further exerting anti-inflammatory effects (Figure 2) [46].

**Figure 3:**
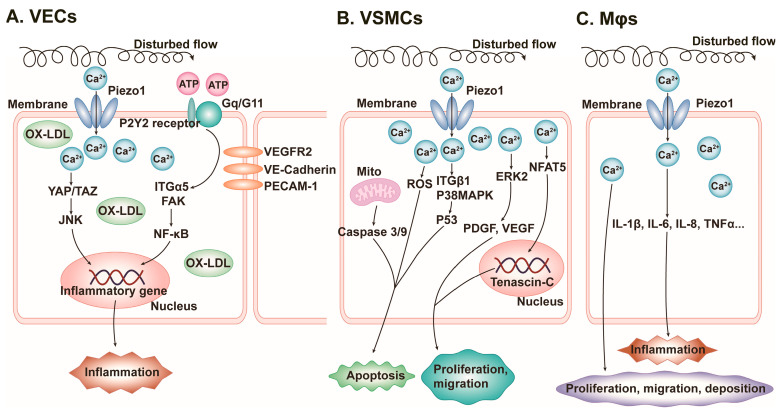
Schematic diagram of the mechanisms by which PIEZO1 promotes atherosclerosis (AS). (**A**) PIEZO1 in vascular endothelial cells (VECs). FAK: focal adhesion kinase, ITGα5: integrin alpha 5, JNK: c-Jun N-terminal kinase, NF-κB: nuclear factor-κ-gene binding, OX-LDL: oxidized low-density lipoprotein, TAZ: transcription coactivator with PDZ-binding motif, YAP: yes-associated protein. (**B**) PIEZO1 in vascular smooth muscle cells (VSMCs). ITGβ1: integrin beta 1, Mito: mitochondrion, NFAT5: nuclear factor of activated T-cells, PDGF: platelet-derived growth factor, ROS: reactive oxygen species, VEGF: endothelial growth factor. (**C**) PIEZO1 in macrophages (Mø). IL: interleukin, TNF-α: tumor necrosis factor-α.

In contrast, when PIEZO1 senses turbulent shear, it induces pathological responses such as vascular inflammation, especially in the medial (non-lateral) aortic arch, leading to atherosclerosis (AS) [47,48]. Mechanistically, PIEZO1 forms a mechanotransduction complex with G_q_/G_11_ and PECAM-1 at the EC junction. The β-subunit of G protein may play an important role in the activation of PIEZO1 by shear force [49]. Activation of the complex by disturbed flow shear further triggers downstream integrin α5 and focal adhesion kinase (FAK), subsequently activating the NF-κB pathway and upregulating the expression of AS-related genes (caspase-1, IL-1β, and NLRP3 inflammatory vesicles) (Figure 3) [48,50]. Furthermore, oxidized low-density lipoprotein (OX-LDL) not only induces inflammatory proteins (NF-κB, c-Jun N-terminal kinase (JNK), and TNF-α), but also upregulates and activates PIEZO1 in VECs of mice with AS. It leads to the activation of yes-associated protein (YAP)/transcription coactivators with a PDZ-binding motif (TAZ) via Ca^2+^ influx and YAP nuclear translocation [51]. YAP and TAZ are thought to promote the atherogenic inflammatory response, which they achieve by augmenting the JNK signalling pathway, ultimately leading to the formation of AS [52]. The expression of inflammation-related factors (NF-κB, JNK, and TNF-α) was significantly decreased in atheromatous plaques when the PIEZO1 inhibitor GsMTx-4 was used or when PIEZO1 was knocked out [51] (Figure 3). PIEZO1 also mediates abnormal VSMC proliferation, migration, and apoptosis, which are involved in AS progression (Figure 3) [53]. Stretch-activated PIEZO1 induces the nuclear translocation of the nuclear factor of activated T-cells 5 (NFAT5), which in turn promotes the expression of Tenascin-C, which has a variety of functions affecting VSMC proliferation, differentiation, and migration (Figure 3) [54]. Stretch-activated PIEZO1 induces the activation of ERK2 and the upregulation of downstream growth factors (PDGF and VEGF) to promote VSMC migration (Figure 3) [48,50]. Moreover, stretch-activated PIEZO1 induces VSMC apoptosis by activating p38MAPK and integrin β1, further activating p53 to upregulate apoptosis regulators [55]. Additionally, it promotes the excessive accumulation of reactive oxygen species (ROS), activates apoptotic enzymes caspase-3 and caspase-9, and disrupts mitochondrial function, leading to mitochondria-dependent apoptosis (Figure 3) [56]. In Møs, activated PIEZO1 mediates the expression of various inflammatory factors, including IL-1β, IL-6, IL-8, and TNF-α, further exacerbating the inflammatory response [57,58]. Activated PIEZO1 induces the proliferation, migration, and deposition of Møs in plaques, promoting the progression of AS (Figure 3) [58].

In addition, the PIEZO1-mediated inflammatory response in Møs plays a key role in the development of a variety of other vascular diseases. Inflammatory responses centered on Møs often promote vascular regeneration. However, a recent research study found that in mouse hindlimb ischemia (HLI) model-induced peripheral artery disease (PAD), the activation of PIEZO1 in Møs by the enhanced stiffness of the surrounding microenvironment activated downstream CaMKII (calcium/calmodulin-dependent protein kinase II)/ETS1 (ETS proto-oncogene 1) signaling, and the transcriptional inactivation of FGF2 inhibited vascular regeneration and reperfusion, ultimately leading to ischemic injury of the hindlimb [59]. Moreover, PIEZO1 in monocytes and transmural Møs of the abdominal aorta is involved in the progression of calcific aortic valve disease (CAVD) and abdominal aortic aneurysm (AAA) diseases, respectively [60,61]. It was found that during the development of AAA, transmural Møs of the abdominal aorta release Netrin-1 to act on VSMCs, followed by the upregulation of the cytoskeletal cross-linker α-Actinin2 in the VSMCs and progression to a mechanically stiff state, which in turn activates PIEZO1 and downstream calcium signaling in VSMCs. The activated PIEZO1 increased VSMC membrane tension and promoted a pathological proteolytic switch, ultimately contributing to pathological remodeling of the vasculature. However, after antagonizing PIEZO1, both matrix degradation and pathological remodeling of the vasculature were inhibited, and the development of AAA was hampered [61]. Therefore, targeting PIEZO1 may become a promising therapy for inflammation-involved diseases such as AS, PAD, CAVD, and AAA.

### 4.2. Blood Pressure

Blood pressure is critical for tissues with proper blood perfusion. Exquisite real-time regulation is essential to maintain stable blood flow and vascular tone [8,9]. PIEZO1 in mammals is involved in modulating vascular tone, including the relaxation and constriction of mesenteric vessels [62,63]. The elevation of mesenteric vascular blood flow or blood pressure activates PIEZO1 in VECs, contributing to mesenteric artery dilation and stabilization of blood pressure. Mechanistically, this process involves ATP production via the PI3K/AMPK/AKT/eNOS pathway [63] and protein kinase A (PKA)-mediated phosphorylation of eNOS at Ser632 [64], both of which induce the production of vasodilator nitric oxide (NO) (Figure 2). During exercise in adult mice, the gastrointestinal vasculature constricts to redirect blood flow to exercise-related organs, and PIEZO1 induces sympathetic activation and mesenteric vasoconstriction [62]. Activated PIEZO1 mediates the inward flow of Ca^2+^ and Na^+^, depolarizing VECs. In the microvessels, the increase in calcium is transmitted through gap junctions to electrically coupled VSMCs, ultimately leading to vasoconstriction [62]. In addition, VECs adapt to blood pressure changes through a biphasic mechanical stress response, involving transient remodeling of the actomyosin cytoskeleton and VE–calmodulin junctions in VECs driven by PIEZO1-mediated Ca^2+^ inward flow [65]. The homeostasis of blood pressure is also maintained by the baroreflex. Both PIEZO1 and PIEZO2 are highly expressed in the nodose–petrosal–jugular ganglion complex (NPJc) baroreceptor nerve endings and synergistically regulate the modulation of the baroreflex to regulate blood pressure [10]. Mammals with genetic variants in PIEZOs were found to develop defects in baroreflex and aortic depressor nerve activity and underwent dysfunction in blood pressure regulation, ultimately developing unstable hypertension and increased blood pressure variability, consistent with the phenotype of humans suffering from baroreflex failure [10]. However, when PIEZO1 and PIEZO2 were knocked out alone, respectively, the baroreflex was not affected, suggesting that PIEZO1 and PIEZO2s regulate the baroreflex synergistically, which in turn maintains the homeostasis of the body’s blood pressure [10].

**Figure 4:**
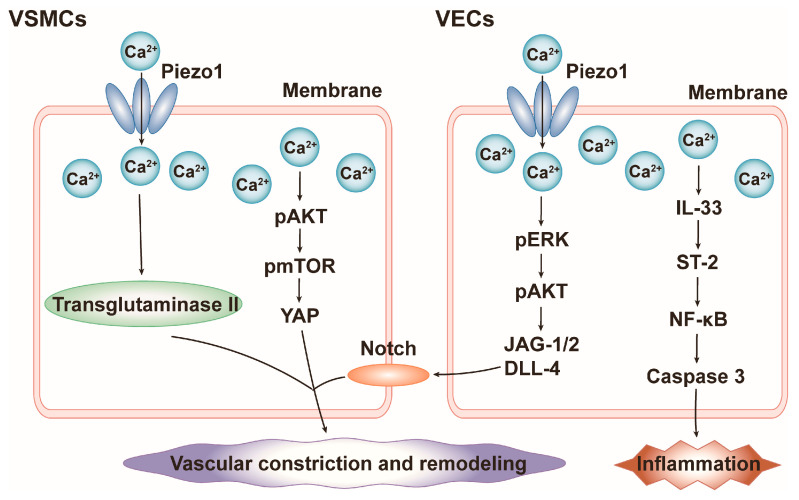
Schematic diagram of the mechanisms by which PIEZO1 promotes vascular remodeling. The left cell diagram represents PIEZO1 in vascular smooth muscle cells (VSMCs); the right cell diagram represents PIEZO1 in vascular endothelial cells (VECs). DLL-4: delta like-4, IL-33: interleukin-33, JAG-1/2: jagged1/2, NF-κB: nuclear factor-κ-gene binding, ST2: suppression of tumorigenicity 2, YAP: yes-associated protein.

However, PIEZO1 highly expressed in VSMCs and VECs of resistance arteries contributes to vascular remodeling and increased blood pressure in pathological states. In a chronic hypertension model induced by angiotensin II (AngII), VSMC-specific PIEZO1 knockout reduces the thickness of the middle layer of blood vessels to alleviate hypertension [66]. Mechanistically, PIEZO1 activated by high blood pressure induces Ca^2+^ inward flow and activates transglutaminase II (a cross-linking enzyme involved in small arterial remodeling), which further leads to cell cytoskeleton and ECM remodeling in smooth muscle cells (Figure 4) [66]. In a model of pulmonary hypertension (PH), PIEZO1 induces the contraction and remodeling of pulmonary arteries by different mechanisms [67]. Mechanistically, in VSMCs, the activation of PIEZO1 mediates extracellular Ca^2+^ inward flow and intracellular Ca^2+^ release and activates the AKT/mTOR/YAP signaling pathway, promoting VSMC contraction and proliferation and facilitating the transition from contractile VSMCs to proliferative ones (Figure 4). In turn, TEAD4, a key transcription factor in the YAP signaling pathway, can upregulate PIEZO1 expression in VSMCs at the transcriptional level [67,68,69,70]. In ECs, upregulated and activated PIEZO1 mediates Ca^2+^ inward flow, which further activates the IL-33/suppression of tumorigenicity 2 (ST2)/NF-κB/Caspase-3 pathway and initiates the inflammatory response (Figure 4). Meanwhile, multiple NF-κB p65 subunit (RELA) binding sites were identified in the promoter region of PIEZO1, and RELA upregulates PIEZO1 expression at the transcriptional level [69,71]. Conversely, it has also been found that Yoda1-activated PIEZO1 promotes endothelial NO-dependent relaxation in early PH rats [72]. In addition, activated PIEZO1-mediated calcium signaling in ECs phosphorylates ERK and AKT, which in turn activates Jagged1/2 (JAG-1/2) and Delta like-4 (DLL-4), and acts on the Notch receptor in VSMCs, inducing the contraction, proliferation, and migration of VSMCs, ultimately resulting in pathologic remodeling of the vasculature (Figure 4) [73]. PIEZO1 is also expressed in renal vascular endothelial cells. Recent reports suggest that PIEZO1-mediated renal vasodilation is the key cause during acute kidney injury caused by acute hyperglycemia-associated hyperosmolarity. Mechanistically, PIEZO1 in renal vascular endothelial cells is activated by hyperglycemia-associated hyperosmolarity, which in turn activates eNOS via CaMKII signaling and AKT signaling, ultimately contributing to the diastolic response of renal arteries and microvessels [74]. Chronic kidney disease, which develops from acute kidney injury, has a complex relationship with heart diseases [75].

### 4.3. Cardiac Hypertrophy

PIEZO1 is expressed in T tubules and intercalated discs of CMs [76,77], where it is activated by mechanical stresses such as blood flow shear and cardiac autonomic pulsation-induced stretch forces to maintain cardiac structural and functional homeostasis [76]. Mechanistically, PIEZO1 in CMs is activated by mechanical stress and mediates Ca^2+^ inward flow. On the one hand, intracellular Ca^2+^ maintains cardiac function through the CICR mechanism; on the other hand, intracellular Ca^2+^ mediates the ROS pathway to stimulate calcium sparks, and downstream Rac1/NOX2 in turn induces ROS production as well as regulates RyR2 (ryanodine receptor 2) activity and calcium sensitivity, thus creating positive feedback, which explains that a low expression of PIEZO1 in CMs can sustainably transduce repeated mechanical stress in the heart to maintain homeostasis [76].

**Figure 5:**
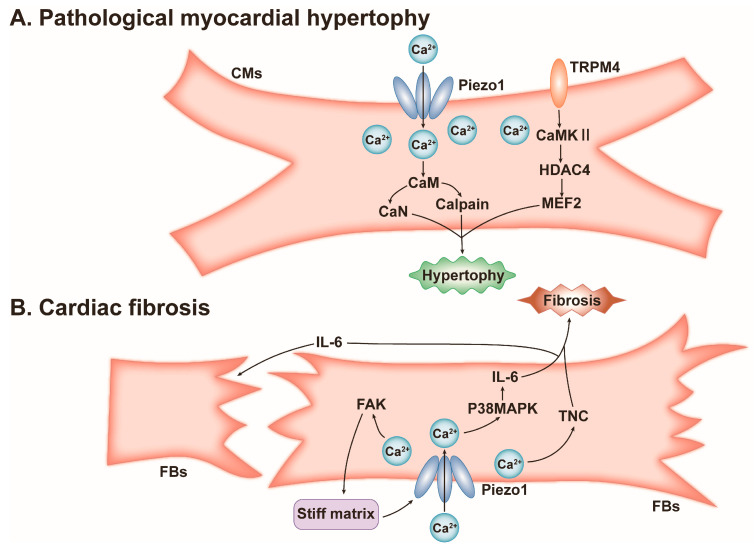
Schematic diagram of the mechanisms by which PIEZO1 promotes pathological myocardial hypertrophy and cardiac fibrosis. (**A**) PIEZO1 in pathological myocardial hypertrophy. CaM: calmodulin, CaMKII: calcium/calmodulin-dependent protein kinase II, CaN: calcineurin, CMs: cardiomyocytes, HDAC4: histone deacetylase 4, MEF2: myocyte enhancer factor 2, TRPM4: transient receptor potential melastatin 4. (**B**) PIEZO1 in cardiac fibrosis. FBs: fibroblasts, FAK: focal adhesion kinase, IL-6: interleukin-6, TNC: tenascin C.

However, when mechanical stress is abnormal, such as in hypertension or when PIEZO1 is abnormal, pathological myocardial hypertrophy can be induced. In in vitro and in vivo experiments, PIEZO1 activated and upregulated by Yoda1 or pathological mechanical traction induces cardiomyocyte hypertrophy. This hypertrophic process is inhibited by the application of PIEZO1 inhibitors (Dooku1 and GsMTx4), PIEZO1 siRNA, and cardiac-specific PIEZO1 knockout [76,77]. Mechanistically, activated PIEZO1 in CMs induces Ca^2+^ inward flow and the disruption of intracellular calcium homeostasis. Intracellular Ca^2+^ binds to the calmodulin (CaM) and activates calcineurin (CaN) and calpain. CaN induces NFAT nuclear translocation and binds to the RCAN1 (hypertrophic transcription factor), and calpain maintains CaN activation by cleaving the autoinhibitory domain of CaN, ultimately leading to myocardial hypertrophy (Figure 5) [77]. Also, PIEZO1 regulates Ca^2+^-activated TRPM4 (transient receptor potential melastatin 4) protein expression in CMs to induce the CaMKII/HDAC4/MEF2 pathway and induce myocardial hypertrophy (Figure 5) [78]. In addition, the expression of PIEZO1 was significantly increased in CMs from hypertrophic cardiomyopathy and adriamycin-induced dilated cardiomyopathy [76,79].

### 4.4. Cardiac Fibrosis

PIEZO1 is highly expressed in human and mouse cardiac fibroblasts (HCFBs and MCFBs). Under physiological conditions, PIEZO1 assumes an anti-fibrotic role. It was found that in adult rat ventricular FBs, PIEZO1 activated by mechanical distraction induced BNP expression and inhibited collagen production, fibroblast proliferation, and activation of the transforming growth factor-β (TGF-β) [80].

However, under pathologically persistent stimuli such as abnormal mechanical stress or inflammatory responses, PIEZO1 mediates the conversion of fibroblasts into myofibroblasts and promotes excessive deposition of myofibroblast-secreted ECM, ultimately leading to fibrosis [79,81]. This fibrotic process is inhibited by cardiac-specific PIEZO1 knockout [77]. Moreover, right ventricular dysfunction and fibrosis induced by PH is associated with the upregulation of PIEZO1 in right ventricular tissue [70]. Mechanistically, activated PIEZO1 in human atrial fibroblasts (HAFBs) directly affects actin assembly by activating FAK, which further leads to increased actin bundle thickness and a more ordered arrangement and promotes cell stiffness. Cell stiffness can increase the secretion of IL-6, and IL-6 transmits the effect of cell stiffness to neighboring fibroblasts via paracrine secretion (Figure 5) [82]. In HCFBs and MCFBs, PIEZO1 activated by Yoda1 mediates Ca^2+^ inward flow and intracellular Ca^2+^ is involved in the p38MAPK/IL-6 pathway of myocardial hypertrophy and fibrosis, but this pathway may be influenced by the matrix or tissue stiffness surrounding PIEZO1 [83,84] (Figure 5). In addition, PIEZO1 activated by Yoda1 may also increase tenascin C (TNC) expression to promote myofibroblast differentiation and ultimately induce myocardial fibrosis (Figure 5) [83].

### 4.5. Heart Failure (HF)

Hypertension causes cardiac overload, which often progresses to pathological myocardial hypertrophy and myocardial fibrosis, ultimately leading to HF. Importantly, PIEZO1-mediated mechanotransduction plays this critical role at each step in this developmental process. Homeostatically, PIEZO1 is highly expressed in a variety of cells in the heart, including ECs, CFs, and CMs, and regulates mechanical adaptation in various HF risk factors such as hypertension, cardiac hypertrophy, and fibrosis [85]. Pathologically, PIEZO1 is also expressed in various immune cells including Møs, dendritic cells, and T-cells. PIEZO1 converts mechanical stimuli into intracellular pro-inflammatory signals, prompting these cells to secrete pro-inflammatory cytokines such as TNF-α, IL-1, and IL-6, which cause myocardial damage and induce HF [85]. In addition, cardiac-infiltrating T-cells expressing PIEZO1 are involved in the remodeling of cardiac lymphatics, disrupting the transport of exuded proteins and lipids, inflammatory and immune responses, and fluid homeostasis, leading to HF [85]. MiR-103a, a potential biomarker of myocardial infarction, can be involved in the progression of hypertension and promote MI and HF by inhibiting PIEZO1 expression [86]. Myocardial infarction (MI) is also a major risk factor for HF. The renin–angiotensin system involved in this process is closely related to PIEZO1. In a rat model of MI-induced HF, PIEZO1 expression was significantly upregulated and was suppressed after the application of losartan (angiotensin II receptor blocker (ARB)) in both in vivo experiments and in vitro experiments [87]. Mechanistically, AngII upregulates PIEZO1 expression in neonatal rat ventricular myocytes via the angiotensin type 1 receptor -Erk1/2 pathway. In addition, MI is often accompanied by arrhythmias. It has been found that cardiac-specific PIEZO1 knockout mice exhibit preserved cardiac function and a low incidence of tachycardia after MI [88]. Mechanistically, PIEZO1-mediated disruption of intracellular calcium homeostasis led to enhanced phosphorylation of RyR2, which further exacerbated calcium leakage and ultimately triggered ventricular arrhythmias. Meanwhile, PIEZO1 activation significantly shortens APD (action potential time duration), induces early afterdepolarization, and enhances triggered activity, leading to arrhythmic remodeling of the cell [89]. Similarly, activated PIEZO1 induces the downregulation of L-type calcium currents in CMs, a major cause of atrial fibrillation (AF). Mechanistically, in atrial-derived HL-1 cells from hypertensive rats, Ca^2+^ inward flow induced by PIEZO1 activated by high mechanical stress decreased L-type calcium currents through the CaM/Src/Pitx2 (paired like homeodomain 2) pathway, which shortened APD and increased AF susceptibility [89]. In addition, a prominent feature of AF-related structural remodeling is fibrosis [90]. It was found that in AFBs from patients with AF, the expression and activity of PIEZO1 were elevated compared to those with a normal sinus rhythm, which corresponded to increased pro-fibrotic IL-6 signaling [90].

## 5. Potential Chemical Therapies Targeting PIEZO1

All of the above research suggests that PIEZO1 may become a potential target for the treatment of cardiovascular diseases in the future. We found that in addition to the agonistic effects of physical–mechanical stress on PIEZO1, several chemical small molecules and endogenous substances can also regulate PIEZO1. However, pharmacological research on PIEZO1 is still in its infancy. We believe that the pharmacological research of PIEZO1 and the development of related regulatory small molecules are significant for the resolution and intervention of PIEZO1.

### 5.1. Agonists

#### 5.1.1. Yoda1

**Figure 6:**
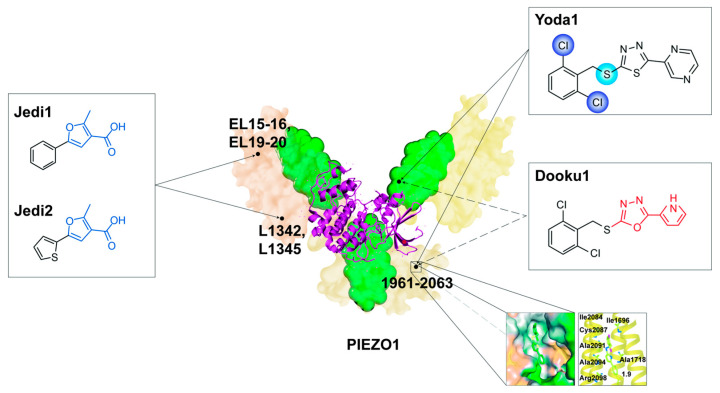
Schematic diagram of active sites and regulators of PIEZO1.

Yoda1, one of the earliest PIEZO1-specific agonists screened from approximately 3.25 million chemical small molecules, has become the most classical chemical regulator for probing PIEZO1 roles in the cardiovascular system [91]. Yoda1 is a highly hydrophobic small molecule compound with high PIEZO1 specificity [91]. Structurally, the dichloro group in the 2,6-dichlorophenyl ring and the thioether linkage are the key structural domains for the binding of Yoda1 to PIEZO1 [92]. Meanwhile, the pyrazine moiety is also a key structural site but is not responsible for binding [92]. Mechanistically, Yoda1 may act on the intracellular blade to bind to a narrow hydrophobic pocket near PIEZO1 residues 1961–2063 in the form of a molecular wedge (Figure 6) [93], contributing to the uncoupling of the coupled structure and increased arm flexibility of PIEZO1, ultimately decreasing the mechanical activation threshold of PIEZO1 and increasing PIEZO1 sensitivity [93,94], as well as prolonging the opening time and shortening the closing time of PIEZO1 channels [95]. Thus, Yoda1 activates PIEZO1 both independently and in concert with external mechanical stress. The activation of PIEZO1 by Yoda1 can be influenced by external factors such as temperature, voltage, and energy regulation, in addition to its own structural properties [95]. The effect of Yoda1 is maximal at room temperature and negative voltage. In contrast, the effect of Yoda1 is attenuated at mammalian body temperature or at depolarizing potentials. In addition, cysteine bridges can inhibit the mechanical and Yoda1 activation of PIEZO1 through energy modulation [95]. Therefore, the exploration of Yoda1 could help develop more modulators of PIEZO1.

#### 5.1.2. Jedi1 and Jedi2

Jedi1 and Jedi2 are two new PIEZO1-specific agonists discovered after Yoda1. They have high PIEZO1 specificity but low affinity [8,14,92]. Jedi1 and Jedi2 contain a common structure furan-3-carboxylic acid moiety, which may be responsible for the activation of PIEZO1 [92]. The mechanism of PIEZO1 activation by Jedi1 and Jedi2 is different from that of Yoda1. Jedi1 and Jedi2 activate PIEZO1 by acting on the extracellular blade to bind to PIEZO1, which can act synergistically with Yoda1. Extracellular loops (EL) 15–16 and EL19–20 on the blade and L1342 and L1345 on the beam are critical for the activation of Jedi1 and Jedi2, but they are not binding sites for PIEZO1 (Figure 6). Jedi1 and Jedi2 and Yoda1 may activate PIEZO1 by acting on different trajectories along the blade–beam pathway. In terms of activation effects, Jedi1 and Jedi2 also modulate PIEZO1 by increasing PIEZO1 mechanosensitivity and slowing inactivation, but with faster activation onset and faster decay than Yoda1 [14,92]. The specific binding sites of Jedi1 and Jedi2 and the overall regulatory mechanisms need to be further investigated and revealed.

### 5.2. Competitive Inhibitors

#### 5.2.1. Dooku1

Dooku1 is a synthetic analogue of Yoda1 formed by reconstituting the pyrazine moiety of Yoda1 into a pyrrole moiety. Dooku1 retains the structure that binds to PIEZO1 but has no effect on PIEZO1 activity (Figure 6), which forms a competitive inhibition with Yoda1 [8,92,93]. Therefore, Dooku1 is often used as a competitive inhibitor of Yoda1 to inhibit PIEZO1 activation in cardiovascular system research; for example, in isometric tension experiments of isolated murine thoracic aortic rings, Dooku1 acted to impede Yoda1-induced aortic relaxation by inhibiting Yoda1 activation of PIEZO1 [96].

#### 5.2.2. Tubeimoside I (TBMS1) and Salvianolic Acid B (SalB)

TBMS1 is screened from traditional Chinese medicine using Yoda1 in 92 small molecules [97]; SalB is a major component of Salvia miltiorrhiza (Danshen) [98]. They are similar to Dooku1 and may inhibit PIEZO1 through a mechanism of competitive inhibition of Yoda1 [97,98]. TBMS1 and Danshen/SalB have been used in cardiovascular research; for example, they can inhibit Yoda1-induced relaxation of the aortic rings [97,98] and SalB can improve the process of AS [98,99].

### 5.3. Non-Competitive Inhibitors

#### 5.3.1. GsMTx-4

GsMTx-4 is a ~4 kDa peptide toxin that was originally extracted in the venom of tarantulas. It can nonspecifically block mechanosensitive cation channels [92,100,101]. Like Yoda1, GsMTx-4 has been widely used as an inhibitor of PIEZO1 in research of the mammalian cardiovascular system [77]. Mechanistically, GsMTx-4 inhibits PIEZO1 through a tension-clamping model, where GsMTx4 binds to the membrane in a membrane tension-dependent manner (not directly on PIEZO1), adjusts the binding pattern through membrane tension changes, and ultimately dissolves membrane tension near PIEZO1 to inhibit PIEZO1, and this inhibition can be reversed by an increase in membrane stress [8,100,101].

#### 5.3.2. Ruthenium Red (RR), Amyloid β (Aβ), and Gadolinium (Gd^3+^)

RR (a polymeric cation), Aβ (an amphipathic macromolecule), and Gd^3+^ (a trivalent lanthanide) all have non-specific inhibition of MS channels. Gd^3+^ is less selective and has less effective inhibition effects compared to GsMTx4 [102]. RR may inhibit PIEZO1 by blocking the central pore channel mechanism [92,103]. Aβ may regulate PIEZO1 by regulating membrane tension and the cytoskeleton [92,104].

### 5.4. Dietary Lipids

Cell membrane and intracellular lipids, as well as lipid metabolism can regulate PIEZO1 activity [105,106]. Dietary polyunsaturated fatty acids have been found to reduce the orderliness and bending stiffness of cell membranes, which affects the inactivation process of activated PIEZO1, whereas saturated fatty acids have the opposite effect on cell membranes, increasing the mechanical threshold for PIEZO1 activation [105]. In addition, cholesterol on the cell membrane can modulate PIEZO1 by stiffening and thickening the cell membrane [106] and acting on STOML3 (a PIEZO1-related binding protein) [107,108]. In VECs, neutral sphingomyelinases, represented by SMPD3 (sphingomyelin phosphodiesterase 3), can maintain PIEZO1 activity by altering the lipid environment surrounding PIEZO1 [109].

### 5.5. Others

There are various other endogenous factors that regulate PIEZO1 in the cell membrane. Phosphatidylinositol present in the cell membrane can cross-link with PIEZO1, which maintains the PIEZO1 opening [107]. Transient receptor potential vanilloid subtype (TRPV1) and transmembrane protein 150C (TMEM150C) indirectly regulate PIEZO1 by affecting phosphatidylinositol [110,111]. SERCA2 (sarcoplasmic endoplasmic reticulum calcium ATPase 2) can reduces PIEZO1 mechanosensitivity and prevents the disruption of intracellular calcium homeostasis [112]. The matrix protein COMP (cartilage oligomeric matrix protein) interacts through the endogenous activation of PIEZO1 to maintain cardiovascular homeostasis, particularly to improve vascular blood pressure [113].

## 6. Challenges and Prospects

Although emerging evidence promotes the understanding of PIEZO1 in cardiovascular physiology and pathology, several challenges remain to be further elucidated. One major challenge is how to improve the detection method for PIEZO1, which is a large molecular weight protein. Conventional ways, such as immunostaining or western blots, may raise the concern of false positive results and it is difficult to show the recycling of PIEZO1 using these. Another challenge is to identify the functional domain of PIEZO1 under distinct conditions. The comprehensive structure of PIEZO1 protein is still not fully unveiled. Small chemical molecules used to study its mechanisms have low affinity and solubility, which limits the in vivo application. Last but not least, the potential side effects of PIEZO1 inhibition remain to be addressed, as PIEZO1 is expressed in various cell types. Cell-type-specific or tissue-specific PIEZO1 inhibition is in great need. Addressing these challenges requires continuous efforts to fully understand the molecular basis and regulatory mechanisms of PIEZO1.

It is noteworthy that along with the gradual deepening of the research on PIEZO1, more and more research techniques on PIEZO1 have been generated. Currently, CRISPR-based genome editing and tracer technology give a great opportunity to detect PIEZO1, tagging it with a flag or fluorescence [23,76,114]. Another promising way is to construct a novel biosensor for SPT (single particle tracking), which has successfully detected the conformation and activation state of target protein [115]. Applying this technique to the observation of conformational changes during the activation of PIEZO1 holds great promise for studying the link between the structure of PIEZO1 and the activation mechanism. The construction of PIEZO1-specific knockout mice models, which also involves CRISPR-based genome editing technology, deserves our attention. For example, PIEZO1^fl/fl^ (floxed PIEZO1) mice crossed with Myh6-Cre/MLC2v (myosin light chain 2 v)-Cre transgenic mice can construct cardiac-specific PIEZO1 knockout mice [76,77]. PIEZO1^fl/fl^ mice crossed with LysM-Cre transgenic mice can construct myeloid cell-specific PIEZO1 knockout mice [59]. These PIEZO1-specific knockout models provide a good technical avenue for us to explore the function and mechanism of PIEZO1 in vivo.

## 7. Conclusions

PIEZO1 is a highly conserved cation channel that plays an essential role as a novel mechanosensor in the cardiovascular system. It converts distinct physical forces into electrical and biochemical signals to promote cardiovascular development and embryonic viability, directing vascular neogenesis, the orderly expansion of the vascular network and the formation of cardiac ventricular structures. It also regulates anti-inflammatory and blood pressure processes in the vasculature and pulsatile pumping and anti-fibrotic processes in the heart to maintain cardiovascular homeostasis. However, under pathological conditions, the aberrant expression or activation of PIEZO1 leads to adverse cardiac remodeling and eventually heart failure. Researchers have explored a variety of pharmacological small molecule compounds or endogenous substances targeting PIEZO1, paving the way for clinical translation.

Given the above research evidence on PIEZO1 as a mechanical sensor in cardiovascular events, we believe that PIEZO1 plays a crucial and bidirectional role in cardiovascular events. Under physiological conditions, PIEZO1 often plays a positive role in maintaining development and homeostasis, but when pathological conditions occur, the excessive activation of PIEZO1 often leads to its effect becoming negative. PIEZO1 is expected to become an emerging target for the prevention and treatment of cardiovascular diseases. However, in the disease prevention stage, intervention with PIEZO1 should be cautious, as the normal activation of PIEZO1 also maintains normal cardiovascular function. In addition, PIEZO1 is expressed and plays a role in many organs, and the side effects of non-targeted organ interventions for PIEZO1 also need to be overcome.

## Abbreviations

AAA	abdominal aortic aneurysm	mPIEZO1	mouse PIEZO1
Aβ	amyloid β	MS	mechanosensitive
ADAM10	Ca^2+^-dependent transmembrane abscission enzyme	MT1-MMP	membrane-type-1 matrix metalloproteinases
AF	atrial fibrillation	Møs	macrophages
AngII	angiotensin II	NFAT5	nuclear factor of activated T-cells 5
APD	action potential time duration	NF-κB	nuclear factor-κ-gene binding
ARB	angiotensin II receptor blocker	NPJc	nodose-petrosal-jugular ganglion complex
AS	atherosclerosis	OH	outer helix
CaM	calmodulin	OX-LDL	oxidized low-density lipoprotein
CaN	calcineurin	PAD	peripheral artery disease
CaMKII	calcium/calmodulin-dependent protein kinase II	PDGF	platelet-derived growth factor
CAVD	calcified aortic valvular disease	PH	pulmonary hypertension
CED	C-terminal extracellular domain	PIEZO1^fl/fl^	floxed PIEZO1
CMs	cardiomyocytes	Pitx2	paired like homeodomain 2
COMP	cartilage oligomeric matrix protein	PKA	protein kinase A
CTD	C-terminal domain	PRP	platelet-rich plasma
DLL-4	delta like-4	RBPJ	recombination signal binding protein for immunoglobulin kappa J region
E	embryonic day	ROS	reactive oxygen species
ECM	extracellular matrix	RR	ruthenium red
ECs	endothelial cells	RyR2	ryanodine receptor 2
EL	extracellular loops	SalB	salvianolic acid B
ETS1	ETS proto-oncogene 1	SERCA2	sarcoplasmic endoplasmic reticulum calcium ATPase 2
FAK	focal adhesion kinase	SMPD3	sphingomyelin phosphodiesterase 3
FBs	fibroblasts	SNPs	single nucleotide polymorphisms
Gd^3+^	gadolinium	SPT	single particle tracking
HAFBs	human atrial fibroblasts	ST2	suppression of tumorigenicity 2
HCFBs	human cardiac fibroblasts	TAZ	transcription coactivator with PDZ-binding motif
HDAC4	histone deacetylase 4	TBMS1	tubeimoside I
HF	heart failure	TGF-β	transforming growth factor-β
HLI	hindlimb ischemia	THBD	thrombomodulin
hPIEZO1	human PIEZO1	TM	transmembrane
hUC-MSCs	human umbilical cord mesenchymal stem cells	TMEM150C	transmembrane protein 150C
ICAM-1	intercellular cell adhesion molecule-1	TNC	tenascin C
IH	inner helices	TNF-α	tumor necrosis factor-α
IL	interleukin	TRPM4	transient receptor potential melastatin 4
ITG	integrin	TRPV1	transient receptor potential vanilloid subtype
JAG-1/2	jagged1/2	VCAM-1	vascular cell adhesion molecule-1
JNK	c-Jun N-terminal kinase	VE-cadherin	vascular endothelial cadherin
KLF	kruppel-like factor	VEGF	vascular endothelial growth factor
MCFBs	mouse cardiac fibroblasts	VEGFR2	vascular endothelial growth factor receptor 2
MEF2	myocyte enhancer factor 2	VECs	vascular endothelial cells
MI	myocardial infarction	VSMCs	vascular smooth muscle cells
Mito	mitochondrion	NO	nitric oxide
MLC2v	myosin light chain 2v	YAP	yes-associated protein
